# Socioeconomic Deprivation and Patterns of Crisis Mental Health Service Utilisation: A Ward-Level Analysis of a London Home Treatment Team

**DOI:** 10.1192/bjo.2026.11641

**Published:** 2026-06-30

**Authors:** Mahnoosh Sanati, Allerdiena Hubbeling

**Affiliations:** 1 City St George's University of London, London, United Kingdom; 2 South West London and St. George’s Mental Health NHS Trust, London, United Kingdom

## Abstract

**Aims:**

Home Treatment Teams, first established in England in 2000, play a key role in delivering community-based psychiatric care. Socioeconomic deprivation has previously been linked with an increased risk of mental illness and poorer health outcomes. However, there is limited local-level research examining how deprivation shapes referral patterns and service utilisation.

This study aimed to investigate the association between socioeconomic deprivation and referral patterns to the Wandsworth Home Treatment Team at the ward level.

It was hypothesised that wards with greater deprivation would have higher referral rates.

**Methods:**

A quantitative retrospective observational study was conducted using anonymised, routinely collected patient data from the Wandsworth Home Treatment Team for 2024. The dataset comprised 801 patients, of whom 685 were included in the analysis following exclusion of individuals residing outside the London Borough of Wandsworth. Ward-level population data were obtained from the 2021 Census.

Referral rates were calculated for each ward and standardised per 1,000 population. Socioeconomic deprivation was measured using a population-weighted average Index of Multiple Deprivation (IMD) score calculated from IMD deciles across the 22 wards of Wandsworth. Pearson’s correlation analysis was performed to examine the association between ward-level deprivation scores and referral rates.

**Results:**

Referral rates varied across wards within the London Borough of Wandsworth, ranging from approximately 0.9 per 1,000 population in the least deprived wards to 3.7 per 1,000 population in the most deprived wards. Lower ward-level IMD scores (reflecting greater socioeconomic deprivation) were associated with higher referral rates, demonstrating a strong negative correlation (Pearson’s *r*=-0.789, p <0.001).

**Conclusion:**

The strong association between socioeconomic deprivation and higher Home Treatment Team referral rates underscores the importance of understanding how socioeconomic determinants and environmental context shape mental health need and service utilization. Integrating deprivation-informed approaches into both patient management and local service planning within psychiatric care frameworks may optimise resource allocation, enhance responsiveness to community-level mental health needs, and ultimately improve patient outcomes.

